# Unveiling age-independent spectral markers of propofol-induced loss of consciousness by decomposing the electroencephalographic spectrum into its periodic and aperiodic components

**DOI:** 10.3389/fnagi.2022.1076393

**Published:** 2023-01-18

**Authors:** Sophie Leroy, Sebastian Major, Viktor Bublitz, Jens P. Dreier, Susanne Koch

**Affiliations:** ^1^Department of Anesthesiology and Operative Intensive Care Medicine (CCM, CVK), Charité—Universitätsmedizin Berlin, Corporate Member of Freie Universität Berlin, Humboldt-Universität zu Berlin, Berlin, Germany; ^2^Center for Stroke Research Berlin, Charité—Universitätsmedizin Berlin, Corporate Member of Freie Universität Berlin, Humboldt-Universität zu Berlin, Berlin, Germany; ^3^Department of Experimental Neurology, Charité—Universitätsmedizin Berlin, Corporate Member of Freie Universität Berlin, Humboldt-Universität zu Berlin, Berlin, Germany; ^4^Department of Neurology, Charité—Universitätsmedizin Berlin, Corporate Member of Freie Universität Berlin, Humboldt-Universität zu Berlin, Berlin, Germany; ^5^Bernstein Center for Computational Neuroscience Berlin, Berlin, Germany; ^6^Einstein Center for Neurosciences Berlin, Berlin, Germany

**Keywords:** loss of consciousness, propofol, anesthesia induction, Electroencephalography, aperiodic activity, periodic activity, ageing, 1/f

## Abstract

**Background:**

Induction of general anesthesia with propofol induces radical changes in cortical network organization, leading to unconsciousness. While perioperative frontal electroencephalography (EEG) has been widely implemented in the past decades, validated and age-independent EEG markers for the timepoint of loss of consciousness (LOC) are lacking. Especially the appearance of spatially coherent frontal alpha oscillations (8–12 Hz) marks the transition to unconsciousness.

Here we explored whether decomposing the EEG spectrum into its periodic and aperiodic components unveiled markers of LOC and investigated their age-dependency. We further characterized the LOC-associated alpha oscillations by parametrizing the adjusted power over the aperiodic component, the center frequency, and the bandwidth of the peak in the alpha range.

**Methods:**

In this prospective observational trial, EEG were recorded in a young (18–30 years) and an elderly age-cohort (≥ 70 years) over the transition to propofol-induced unconsciousness. An event marker was set in the EEG recordings at the timepoint of LOC, defined with the suppression of the lid closure reflex. Spectral analysis was conducted with the multitaper method. Aperiodic and periodic components were parametrized with the FOOOF toolbox. Aperiodic parametrization comprised the exponent and the offset. The periodic parametrization consisted in the characterization of the peak in the alpha range with its adjusted power, center frequency and bandwidth. Three time-segments were defined: preLOC (105 – 75 s before LOC), LOC (15 s before to 15 s after LOC), postLOC (190 – 220 s after LOC). Statistical significance was determined with a repeated-measures ANOVA.

**Results:**

Loss of consciousness was associated with an increase in the aperiodic exponent (young: *p* = 0.004, elderly: *p* = 0.007) and offset (young: *p* = 0.020, elderly: *p* = 0.004) as well as an increase in the adjusted power (young: *p* < 0.001, elderly *p* = 0.011) and center frequency (young: *p* = 0.008, elderly: *p* < 0.001) of the periodic alpha peak. We saw age-related differences in the aperiodic exponent and offset after LOC as well as in the power and bandwidth of the periodic alpha peak during LOC.

**Conclusion:**

Decomposing the EEG spectrum over induction of anesthesia into its periodic and aperiodic components unveiled novel age-independent EEG markers of propofol-induced LOC: the aperiodic exponent and offset as well as the center frequency and adjusted power of the power peak in the alpha range.

## Introduction

General anesthesia is commonly induced with an opiate followed by a bolus of propofol, a positive modulator of GABA_A_-receptor mediated inhibition (Trapani et al., 2000). Until today, to recognize the timepoint of loss of consciousness (LOC) anesthesiologists rely on clinical signs like the suppression of brain stem reflexes or missing response to verbal or pain stimuli. Spectral dynamics of propofol-induced LOC have been extensively described in the frontal electroencephalograms (EEG) of non-geriatric patients (Kuizenga et al., 2001; Boly et al., 2012; Purdon et al., 2013). However, a major share of general anesthesia is conducted on elderly and vulnerable patients, who are most prone to develop neurocognitive complications (Staheli and Rondeau, 2022).

Although still insufficiently understood, the transition to unconsciousness represents a key moment for the postoperative outcome of elderly patients (Koch et al., 2021; Windmann et al., 2022). Propofol causes a non-physiological disbalance in the inhibitory inputs of GABAergic neurons on pyramidal cells. Subcortical circuits induce a radical re-organization in cortical networks (Ching et al., 2010; Hutt and Longtin, 2010; Brown et al., 2011; Moody et al., 2021). A switch occurs from a high frequency low amplitude spectrogram to high amplitude low frequency oscillations (Purdon et al., 2015b). Alpha oscillations migrate in the scalp EEG from the occipital to the frontal channels in a process called anteriorization. High-density EEG recordings demonstrate that propofol-induced frontal alpha waves are spatially coherent, possibly due to the anesthetics’ effect on thalamocortical loops (Ching et al., 2010; Cimenser et al., 2011). Overall, the predominance of spatially coherent frontal alpha oscillations superimposed on delta and slow waves is a recurrently proposed spectral power distribution to recognize the unconscious state (Cantero et al., 1999; Gugino et al., 2001; Purdon et al., 2013).

The EEG spectrum can be decomposed into a periodic and an aperiodic component. The superimposed periodic component corresponds to coordinated rhythmic activity around a frequency value, often summarized in classical power bands: sub-delta, delta, theta, alpha, beta, and gamma. Periodic oscillations detectable by scalp EEG arise from cortical neural populations synchronized by common, subcortical generators (Pascual-Marqui et al., 2009).The underlying aperiodic component corresponds to asynchronous spectrum-wide neural rhythms(Miller et al., 2009; Donoghue et al., 2020). Long considered as noise, aperiodic activity delivers information about cerebral activity states with clinical correlates. Changes in aperiodic activity have been described in physiological processes like sleep (Lendner et al., 2020) and ageing (Voytek et al., 2015) but also in neurological (Lanzone et al., 2022; Zhang et al., 2022) and psychiatric disorders (Arnett et al., 2022; Manyukhina et al., 2022). Aperiodic parametrization has been undertaken in non-geriatric patients under general anesthesia: propofol-induced unconsciousness engenders an increase in the aperiodic activity (Brake et al., 2021).

In this explorative analysis we decomposed the EEG spectrum around propofol-induced loss of consciousness into its periodic and aperiodic components in a young and an elderly cohort. Our goal was to find markers of the dynamic transition to unconsciousness in the EEG. Furthermore, we examined cohort differences in the spectral properties to shed light on the age-dependent divergences.

## Materials and methods

### Trial protocol

This analysis is a subproject of the ACDC-Trial (NCT04320082), which was approved by the local ethics committee (Charité Universitätsmedizin Berlin, EA2/029/20). Written approval was obtained from all participants according to the Declaration of Helsinki. Ethical and scientific quality standards were respected following the ICH-GCP guidelines. Patients undergoing general anesthesia for elective orthopedic surgery lasting more than 60 min at the Charité Universitätsmedizin Berlin, Campus Virchow Klinikum were enrolled in this prospective observational trial. Patients with preexisting psychiatric or neurological diseases as well as patients taking centrally acting medication were excluded.

Two age cohorts were formed: a young cohort including 9 patients between 18 and 30 years old, and an elderly cohort with 14 patients over 70 years old. Clinical data were collected including age, sex, height, weight, physical status in the classification system of the American Society of Anesthesiologists (ASA-Score), concomitant diseases, and medication.

Premedication and anesthesia were conducted following the standard operation procedures of the clinic (Spies, 2005). Anesthesia was induced intravenously with a fentanyl bolus followed by a bolus of propofol. The anesthetic doses were individually defined by the anesthesiologist following the clinical presentation of the patients (age, weight, sex, and concomitant diseases). Propofol was infused manually, without a syringe driver, by a nurse anesthetist. During induction of anesthesia the lid closure reflex was regularly tested to estimate the timepoint of LOC. A non-depolarizing neuromuscular blocker was given to relax the airways if endotracheal intubation was indicated. After a stable sedated state was reached patients were either intubated with an endotracheal tube or a laryngeal mask airway was introduced. Patients with early postoperative neurocognitive disorders were excluded from this analysis.

### Data analysis

Twenty-one Ag/AgCl electrodes (EasyCap GmbH, Woerthsee-Etterschlag, Germany) were placed preoperatively following the 10/20-system on the scalp. The reference and ground electrodes were placed at AFz and FCz, respectively. A full-band, direct current (DC)/alternating current (AC)-EEG reaching from 0 to 5,000 Hz was recorded with a BrainAmp DC amplifier and BrainVision Recorder (Brain Products GmbH, Gilching, Germany). Impedances were kept below 5 kOhm. An event marker was set in the EEG recordings at LOC, defined as the loss of lid closure reflex. Event markers were also set at the start of propofol infusion and at intubation.

**Figure 1:**
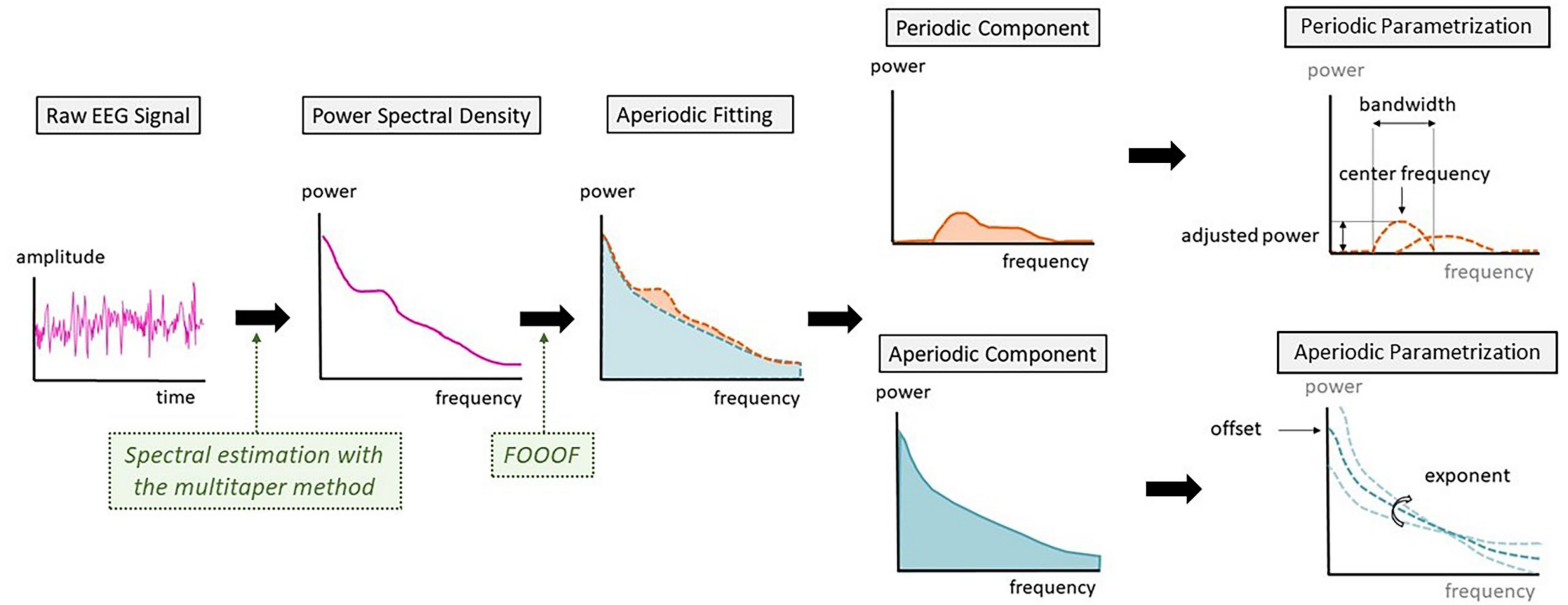
Schematic representation of the steps between raw electroencephalography (EEG) signal and the parametrization into periodic and aperiodic components.

EEG recordings were aligned at the LOC event and cut to six-minute episodes from 120 s before to 240 s after LOC. 30 s EEG segments were defined for statistical analysis: preLOC (105–75 s before LOC), LOC (15 s before to 15 s after LOC), postLOC (190–220 s after LOC, Figure 1). The segmentation of the EEG was based on the clinical markers of anesthesia. The preLOC segment represented the time shortly before propofol induction. The LOC segment comprised the 30 s around the loss of lid closure reflex. The postLOC segment depicted the very early sedation state around the start of mechanic ventilation.

### Spectral analysis

Spectral analysis was performed with the Chronux toolbox (version 2.12 v03)^1^ in MATLAB (MATLAB R2022a, MathWorks Inc.; Partha Mitra, 2009). Preprocessing of the EEG included trendline removal, down-sampling to 500 Hz and bandpass filtering (0.1–45 Hz). EEGs were visually inspected regarding the occurrence of burst-suppression patterns. Power spectrum was estimated using the multitaper method with the following parameters: moving window length of 2 s and shift 0.1 s, time-bandwidth product of 2 and number of tapers of 3. The frequency bands were defined as followed: sub-delta (0.1–1.5 Hz), delta (1.5–4 Hz), theta (4–8 Hz), alpha (8–12 Hz), beta (12–30 Hz) and gamma (30–45 Hz). A spectrogram was computed and normalized by the total power at each timepoint of the spectrogram for one frontal channel (Fp1). Power Spectral Density (PSD) arrays were computed for each time-segment (preLOC, LOC, and postLOC) from the non-normalized spectra of the respective age-group.

The analysis of the aperiodic components in the spectral power distribution was computed at each timepoint with the Fitting Oscillations and One-Over-f (FOOOF) toolbox (version 1.0.0, Donoghue et al., 2020) in MATLAB and averaged over the time segments with the following settings: peak width limit 0.5–12 Hz, infinite maximum number of peaks, minimum peak height of 0 *μV*^2^, peak threshold of 2 standard deviations and a fixed aperiodic mode without a knee parameter.

Characterization of the aperiodic activity comprises the offset and exponent, respectively the y-intercept and the slope of the decay (Donoghue et al., 2020). A flatter exponential curve corresponds to a smaller exponent value and a steeper curve to a greater exponent value.

By deducting the aperiodic fit to the raw PSD, the periodic power peaks over the aperiodic fit were extracted for both age groups. These periodic power peaks were represented in spectrograms. When negative values were reached due to aperiodic overfitting, values were replaced with zeros for the power peak spectrograms.

The FOOOF toolbox also parametrizes the periodic peaks detected by the algorithm. Given the dynamics of alpha oscillations over the transition to unconsciousness, we analyzed the characteristics of the alpha peak. For every patient, we searched if a peak of power with a center frequency between 8 and 12 Hz was present. These were defined as alpha peaks and were further described by differentiating their center frequency, the adjusted power over and above the aperiodic component and the bandwidth of the oscillation peak (Figure 1).

### Statistical analysis

Statistical analysis was carried out in IBM SPSS Statistics (Version: 28.0.1.0). Statistical significance in clinical data was assessed with the appropriated test (Chi-squared test, *t*-test, or Mann–Whitney-*U* test). Except for the assessment of differences in the population demographics, results were not interpreted as significant or not significant given the unavoidable multiple testing in an explorative pilot study design. To minimize type I and type II errors in statistical interpretation, we defined a reporting level of *p* ≤ 0.05 without correcting for multiple testing.

The following EEG parameters were averaged over the time-segments: aperiodic parameters (exponent, offset), alpha peak characteristics (center frequency, adjusted power, and bandwidth). We assessed differences between the groups in these parameters with a repeated-measures ANOVA with timepoints and EEG parameters as within-subjects factors and the age group as between-subjects factor.

Using a custom-written MATLAB code, we assessed differences in the density spectral array with a Mann–Whitney-*U* test at each sampling point and each frequency bin in the raw spectrogram and plotted the results in a three-dimensional space. Only results with *p* ≤ 0.05 were displayed, in blue when the power was higher in the young patients and in red when the power was higher in the elderly. A similar method was applied in a paper by Obert and colleagues (Obert et al., 2020). The same procedure was applied to the spectrograms of the aperiodic activity. We visually compared these plots of differences between the raw spectrograms and the aperiodic activity spectrograms.

## Results

### Study population, demographic data, clinical data

Out of the 23 patients included in this analysis, 14 were over 70 years old and 9 were between 18 and 30 years old. The mean age in the young cohort was 25.7 years while the mean age in the elderly cohort amounted to 80.1 years. The BMI was significantly higher in the elderly patients (*p* = 0.037). The patients of the young cohort had significantly smaller ASA-scores (*p* < 0.001). Both groups received similar doses of fentanyl preoperatively (*p* = 0.968). Elderly patients received around half the doses per kg of propofol administered to young patients for induction of anesthesia (*p* < 0.001). The time between the start of the propofol injection and the LOC (*p* = 0.075) as well as the time between LOC and intubation (*p* = 0.710) was similar in the elderly compared to the young cohort (Table 1). Two young and four elderly patients exhibited burst-suppression patterns after induction of anesthesia.

**Table 1:** Characteristics of the study population.

Cohort	Young (value ± SD)	Elderly (value ± SD)	*p*-Value
Age (years)	23.7 ± 3.0	80.1 ± 3.5	–
Sex male/female (%)^1^	100/0	71.4/28.6	0.078
BMI (kg/m2) ^2^	**23.4 ± 3.0**	**28.1 ± 6.6**	**0.037**
ASA-Score I/II/III/IV/V^1^	**8/1/0/0/0**	**0/5/7/2/0**	**<0.001**
Dose fentanyl for induction (μg)^2^	227.8 ± 44.1	228.6 ± 46.9	0.968
Dose propofol for induction (mg/kg)^2^	**3.41 ± 0.63**	**1.78 ± 0.66**	**<0.001**
Time between start propofol injection & LOC^3^ (s)	68.9 ± 14.9	52.6 ± 18.9	0.075
Time between LOC and Intubation/LMA^3^ (s)	151.0 ± 105.2	183.0 ± 120.2	0.71

1 Chi-squared test.

2 *t*-test.

3 Mann–Whitney-*U*-test.

Results with *p*-values below 0.05 were represented bold.

### Descriptive spectral analysis

**Figure 2:**
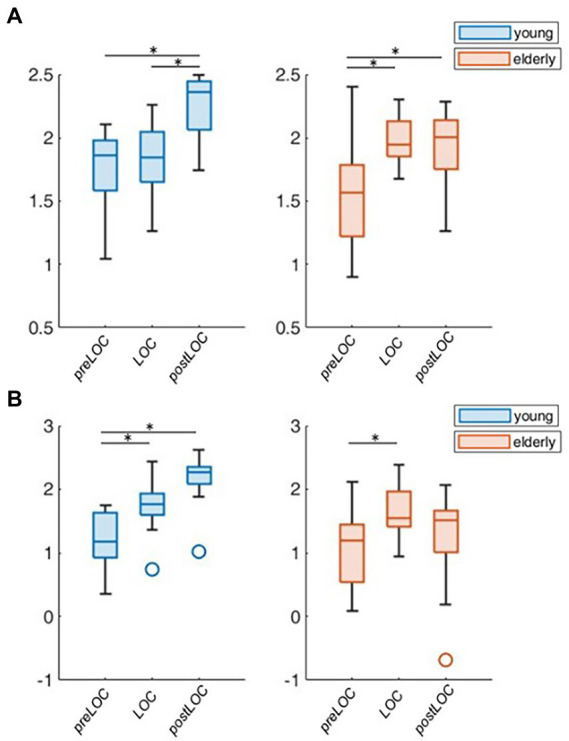
Raw spectrograms and spectrograms of periodic components with group differences. **(A)** Group-averaged raw median spectrograms at Fp1 for the young (upper panel) and elderly (middle panel) cohort with representation of the differences with *p* ≤ 0.05, calculated with Mann–Whitney-*U* test at each timepoint and each frequency bin, in blue when the power is higher in the young patients and red when the power is higher in the elderly. **(B)** Group-averaged median periodic component spectrograms at Fp1 for the young (upper panel) and elderly (middle panel) cohort with representation of the differences with *p* ≤ 0.05, calculated with Mann–Whitney-*U* test at each timepoint and each frequency bin, in blue when the power is higher in the young patients and red when the power is higher in the elderly.

Young patients exhibited a burst in alpha, beta, and low gamma power at LOC in the spectrogram (Figure 2A, upper panel). Inspection of individual spectrograms of this age-group showed that the power burst was present in each patients’ spectrogram and had a uniform dynamic: it appeared in the low gamma, spread to the beta, and finally reached the alpha range. During the early sedation state, increased power values for all frequencies under 30 Hz remained, and strong slow and alpha bands established. The elderly cohort had more heterogeneous raw spectral signatures during the transition to unconsciousness (Figure 2A, middle panel). Upon individual spectrogram analysis, four elderly patients appeared to develop the power burst around LOC like the young patients. The remaining patients either showed an attenuated power increase or no differentiable high frequency power bursts.

**Figure 3:**
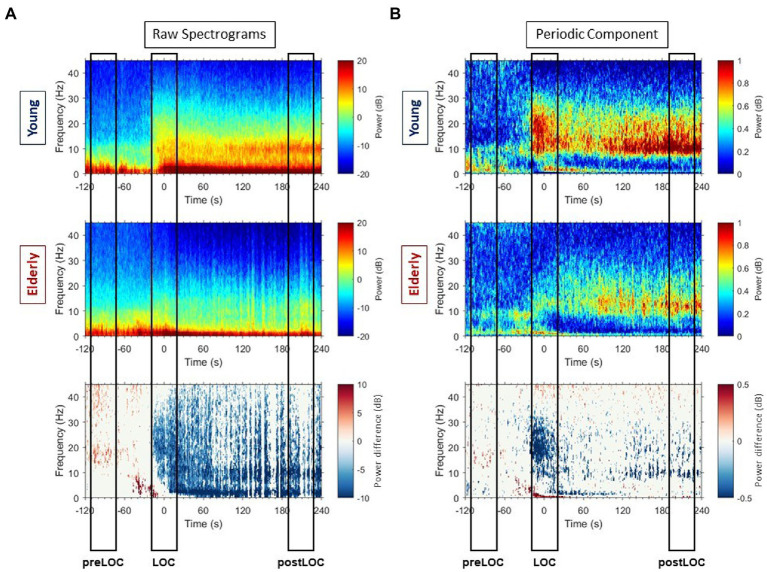
Decomposition of the power spectral density (PSD) array in its periodic and aperiodic components for the young (blue, *n* = 9) and elderly (red, *n* = 14) cohort at Fp1. Left column: raw PSD, Middle column: aperiodic component of the PSD. Right column: periodic component of the PSD at preLOC **(A)**, LOC **(B)**, postLOC **(C)**. Shaded Areas correspond to the interquartile range. Vertical dashed lines mark the limits of the frequency bands.

Overall, the young patients developed higher power values than elderly in all frequencies around LOC in the raw spectrum (Figure 3, left column). Clusters of differences with higher power were unveiled: in the gamma, beta to alpha frequencies around LOC and in the alpha and slow bands after LOC (Figure 2A, lower panel).

### Aperiodic components of the EEG spectrum

We decomposed the spectral activity in its periodic and aperiodic components. With the obtained exponent and offset we built the slope of the aperiodic component (Figure 3, middle column).

**Figure 4:**
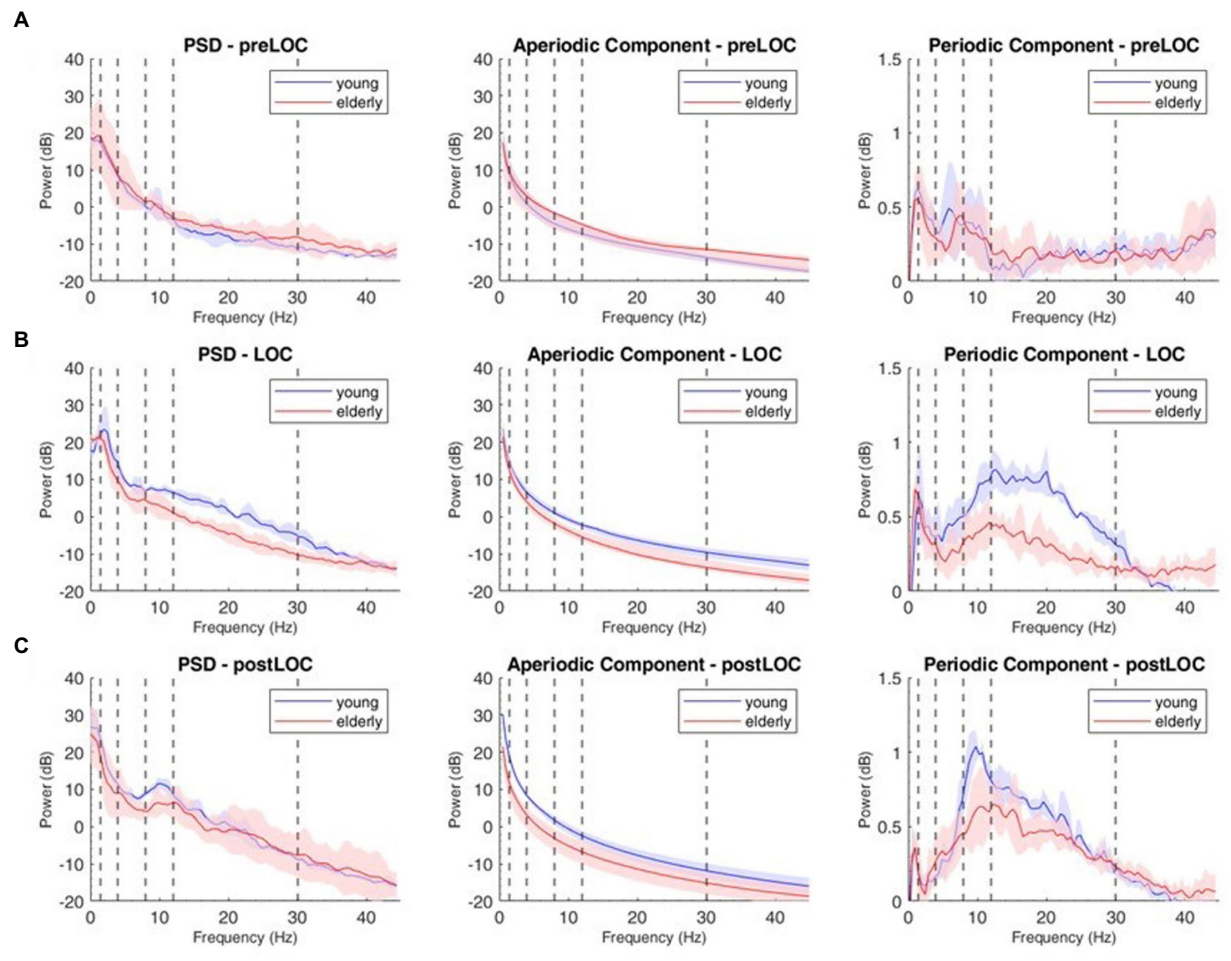
Group-wise boxplots of aperiodic parameters over loss of consciousness (LOC). **(A)** Aperiodic exponent. **(B)** Aperiodic offset. *p*-values ≤ 0.05 were marked with a ^*^.

Overall, the transition to unconsciousness was associated with an increase in the aperiodic exponent in both age groups (young: *p* = 0.004, elderly: *p* = 0.007). However, the elderly cohort already showed the increase between preLOC and LOC (*p* < 0.001) while it occurred between postLOC and LOC in the young cohort (*p* = 0.002; Figure 4A). Upon individual patient plot analysis, 8 of the 9 young patients (89%) as well as 5 of the 14 (36%) elderly patients had a drop in the exponent between preLOC and LOC. The aperiodic offset increased between preLOC and LOC in both groups (young: *p* = 0.020, elderly: *p* = 0.004). The value further increased between LOC and postLOC in the young patients (*p* = 0.001). The exponent and the offset were higher in the young than elderly patients with a statistically discernable difference solely at postLOC (Table 2; Figure 4; Supplementary Table S1).

**Table 2:** Difference between age cohorts at each time-segment with *p*-values computed with repeated-measures ANOVA.

Parameter	Time point	Mean young cohort [95% confidence interval]	Mean elderly cohort [95% confidence interval]	*p*-Value
Aperiodic parametrization				
Exponent	preLOC	1.766 [1.501 2.031]	1.564 [1.352 1.777]	0.231
(*μV*^2^/Hz)	LOC	1.828 [1.632 2.024]	2.003 [1.846 2.160]	0.161
	postLOC	**2.237 [2.031 2.442]**	**1.917 [1.752 2.081]**	**0.019**
Offset	preLOC	1.212 [0.834 1.589]	1.097 [0.795 1.400]	0.628
(*μV*^2^)	LOC	1.733 [1.414 2.052]	1.631 [1.375 1.887]	0.609
	postLOC	**2.141 [1.690 2.593]**	**1.279 [0.917 1.641]**	**0.005**
Periodic parametrization of the alpha peak (8–12 Hz)				
Center frequency	preLOC	10.,068 [9.498 10.638]	9.999 [9.541 10.456]	0.845
(Hz)	LOC	10.962 [10.443 11.481]	11.034 [10.618 11.450]	0.824
	postLOC	10.872 [10.355 11.389]	11.037 [10.623 11.452]	0.610
Adjusted power	preLOC	0.803 [0.667 0.939]	0.795 [0.686 0.904]	0.925
(*μV*^2^)	LOC	**1.060 [0.936 1.186]**	**0.794 [0.694 0.894]**	**0.002**
	postLOC	**1.277 [1.115 1.438]**	**1.058 [0.929 1.188]**	**0.039**
Bandwidth	preLOC	2.217 [1.830 2.604]	2.358 [2.048 2.669]	0.559
(Hz)	LOC	**2.985 [2.663 3.306]**	**2.482 [2.225 2.740]**	**0.019**
	postLOC	3.267 [2.844 3.690]	2.775 [2.435 3.114]	0.073

Results with p-values below 0.05 were represented bold.

### Periodic components of the EEG spectrum

By subtracting the aperiodic slope to the raw PSD, we unveiled the periodic component of the power spectrum (Figure 3, right column). We computed group spectrograms of solely the periodic activity (Figure 2B, upper and middle panel). Most of the clusters of differences from the raw spectrograms did not appear. Solely a cluster in the low gamma, beta and alpha bands around LOC and a cluster through the alpha range after LOC remained frankly discriminable (Figure 2B, lower panel).

**Figure 5:**
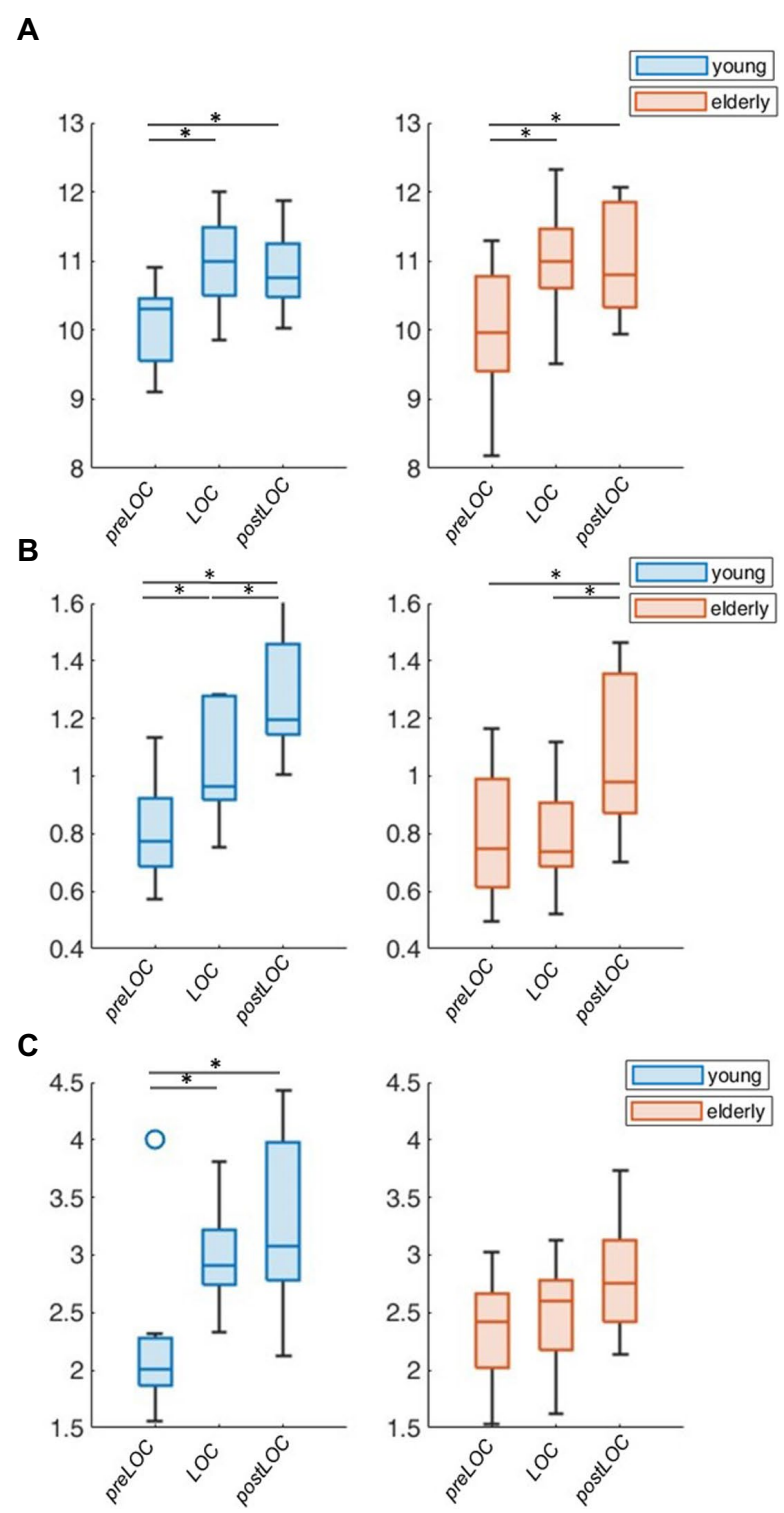
Group-wise boxplots of alpha peak characteristics over LOC. **(A)** Center frequency. **(B)** Adjusted power. **(C)** Bandwidth. *p*-Values ≤ 0.05 were marked with a ^*^.

To explore the group difference in the periodic oscillations in the alpha range, we undertook the further characterization of the peak. Both cohorts showed an acceleration in the center frequency of the alpha peak from around 10 to 11 Hz during the transition to unconsciousness (young: *p* = 0.008, elderly: *p* < 0.001). There were no group differences in the center frequency of the alpha peak. The adjusted power of the alpha peak increased in both cohorts between preLOC and postLOC (young: *p* < 0.001, elderly *p* = 0.011). This power rise started between preLOC and LOC in the young (*p* = 0.002) whereas it appeared between LOC and postLOC in the elderly (*p* = 0.001). Young patients exhibited a higher adjusted alpha power at LOC (*p* = 0.002) and postLOC (*p* = 0.039) than the elderly. The peaks’ bandwidth enlarged with induction of anesthesia in young patients (*p* = 0.002). No clear effect was obvious in the elderly cohort regarding the bandwidth of the alpha peak. Young patients exhibited a larger peak bandwidth than elderly patients solely at LOC (*p* = 0.019; Table 2; Figure 5; Supplementary Table S1).

## Discussion

By decomposing the frontal EEG during the transition to propofol-induced unconsciousness into its periodic and aperiodic components, we unveiled age-independent markers of LOC: an increase in the aperiodic exponent and offset, an acceleration in the center frequency of the periodic alpha peak and an increase in the adjusted power of the alpha peak. We depicted age-related differences in both aperiodic parameters after LOC as well as in the power and bandwidth of the periodic alpha peak during the transition to unconsciousness.

### Electroencephalography-derived markers of LOC

Anesthesia induction resulted age-independently in an increase of the aperiodic exponent. Recent work suggests that the exponent reflects the cortical balance between excitation and inhibition (Bédard and Destexhe, 2009; Gao et al., 2017). The increase possibly reflects the predominance of inhibitory synaptic currents due to the massive propofol-mediated GABAergic activation (Hutt and Longtin, 2010). LOC was also characterized by an increase of periodic activity in the low gamma, beta, and alpha frequencies in both cohorts. This corresponds to the previously described phenomenon of a “traveling peak” (Purdon et al., 2013). Both cohorts exhibited an increase in the adjusted power and an acceleration in the center frequency of the alpha peak during LOC.

It is notable that most young patients as well as some elderly patients exhibited a drop in the exponent between shortly before LOC. This transduces in a flatter slope, possibly induced by a rise of excitatory synaptic activity. Especially young patients frequently developed so-called “paradoxical excitation” during early propofol-induced anesthesia or at low propofol doses for light sedation with preserved brainstem reflexes (Lee et al., 2019). The exact neurophysiological mechanism behind this phenomenon is not sufficiently understood but the exponent value could be an EEG marker of this process.

### Age-induced changes in the power spectrum

Due to neurophysiological and neuroanatomical alterations including brain atrophy (Lee and Kim, 2022), cortical thinning (McGinnis et al., 2011; Fjell et al., 2014), and neurotransmitter disbalance (Mora et al., 2007), EEG recordings undergo characteristic changes with growing age. Elderly patients under general anesthesia are known to exhibit a decreased power in the EEG due to an amplitude reduction of 2- to 3-fold compared to young adults (Purdon et al., 2015a). We did not correct for this effect to discern whether difference arose from changes in periodic or aperiodic components.

When adjusting by the aperiodic fit, elderly patients exhibited similar spectrograms than young patients with an anesthesia-induced increase in the low gamma, beta, and alpha power. This finding implies that a major element of the age-related difference in the power distribution arises from the differences in the aperiodic neural rhythms. According to the neural noise hypothesis, due to a desynchronization of spiking, ageing is associated with an increase in neural background noise activity—measured as a flattening of the slope, a smaller exponent—inducing deficits in neural communication (Hong and Rebec, 2012; Voytek et al., 2015). The resulting decrease in the effective signal-to-noise ratio has been linked to age-related cognitive decline (Salthouse and Lichty, 1985; Voytek et al., 2015).

We also unveiled differences in the overlayed synchronized periodic activity. Elderly patients exhibited a reduced adjusted alpha power already in the first minutes of anesthesia, at a very early sedation state. Decreased alpha power during intraoperative stable sedation is associated with preoperative cognitive impairment (Koch et al., 2019), intraoperative occurrence of burst suppression patterns (Shao et al., 2020) as well as the emergence of a postoperative delirium (Koch et al., 2021).

### Anesthesia induction in the elderly

Despite the commonly carried out reduction of anesthesia doses in geriatric patients, drug overdosing occurs often and mostly during anesthesia induction (Phillips et al., 2015; Akhtar et al., 2016). In our investigation, elderly patients received about half of the propofol doses given to the young adults, as recommended (Rivera and Antognini, 2009). Nevertheless, they were more likely to develop burst-suppression patterns. Burst suppression patterns are an EEG marker of excessive anesthesia depth, pathophysiologically linked to drug-induced hypotension (Georgii et al., 2022). Higher age and ASA physical status ≥ 3 are predictors of hypotension after induction of anesthesia (Reich et al., 2005). Burst suppression patterns are associated with the emergence of postoperative delirium (Soehle et al., 2015; Fritz et al., 2016). We would like to further emphasize the crucial importance of a risk minimization during anesthesia induction. Especially in geriatric and multimorbid patients, care should be given regarding the choice of anesthetic agents, drug doses, and speed of injection (Obert et al., 2020).

Reliable EEG-derived indices parametrizing the highly dynamic transition during LOC are still lacking. Common clinical monitoring devices are not specifically trained for this purpose and are inherently based on algorithms that are updated with a lag of over 30 s (Ferreira et al., 2019). This is sufficient to monitor episodes of steady state anesthesia but not for anesthesia induction. Neuromonitoring-based anesthesia titration has led to a significant decrease in postoperative neurocognitive complications (Radtke et al., 2013). The future of intraoperative neuromonitoring lies in the further evolution towards a personalized precision medicine with real-time EEG-derived indices allowing prompt adjustment of anesthesia.

## Limitations

Due to a high inter-investigator variability and difficult objectification without electromyographic recordings, care should be given when interpreting the event of loss of lid closure reflex. To facilitate the readability of our findings, we called LOC the timepoint of loss of lid closure reflex. This event does not reliably represent the precise timepoint, further underlining the need for an EEG-based objectification of LOC.

We used the FOOOF toolbox to parametrize aperiodic activity. It is established and commonly used to this end (Arnett et al., 2022; Brookshire, 2022; Shuffrey et al., 2022). The FOOOF toolbox delivers an accurate aperiodic parametrization in “easy” PSDs without plateau or major peak overlap (Gerster et al., 2022). We noticed that the aperiodic curve was overfitted in the gamma range of the young patients as negative values were reached in the power peaks. The results of our investigation regarding the aperiodic activity depend on the accuracy of the FOOOF toolbox parametrization.

Given the explorative character of this study and associated unavoidable multiple testing, *p*-values were calculated and listed but not interpreted as significant but rather as statistical aids for interpretation, requiring further studies to confirm the findings (Bender and Lange, 2001). The intention here was to describe these dynamics in a hypothesis-generating manner instead of directly implementable parameters (Hollenbeck and Wright, 2016).

## Conclusion

In this study we identified several age-independent EEG-derived features that concur with propofol induced loss of consciousness. These features can potentially be used to assess the moment of loss of consciousness more precisely and reliably in an age-independent fashion. This may provide tools for an optimization of EEG-based anesthesia induction, a procedure known to pose significant risk to the elderly. The role of age-dependent alterations in the periodic and aperiodic spectral signatures of anesthesia-induced LOC needs to be further examined.

## Data availability statement

The raw data supporting the conclusions of this article will be made available by the authors, without undue reservation.

## Ethics statement

The studies involving human participants were reviewed and approved by Ethikkommission der Charité—Universitätsmedizin Berlin. The patients/participants provided their written informed consent to participate in this study.

## Author contributions

SL, SM, JD, and SK conceived and designed the trial. SL conducted the experiments and wrote the first draft of the manuscript. SL, VB, and SM analyzed the data. All authors contributed to the article and approved the submitted version.

## Funding

JD was supported by Deutsche Forschungsgemeinschaft (DFG DR 323/10-1), and Era-Net Neuron EBio2, with funds from BMBF (01EW2004). SL was financially supported by the Berlin Institute of Health (BIH) with a research stipend. SK was funded by the Deutsche Forschungsgemeinschaft (DFG, German Research Fundation) – Project number KO 4249/3-1).

## Conflict of interest

SK is an inventor on patents, sold to Medtronic, and received speaker’s honoraria from Medtronic.

The remaining authors declare that the research was conducted in the absence of any commercial or financial relationships that could be construed as a potential conflict of interest.

## Publisher’s note

All claims expressed in this article are solely those of the authors and do not necessarily represent those of their affiliated organizations, or those of the publisher, the editors and the reviewers. Any product that may be evaluated in this article, or claim that may be made by its manufacturer, is not guaranteed or endorsed by the publisher.

## Footnotes

^1^ http://chronux.org/
