# Supplementary material for: Unveiling age-independent spectral markers of propofol-induced loss of consciousness by decomposing the electroencephalographic spectrum into its periodic and aperiodic components
Source: Front Aging Neurosci. 2023 Jan 18;14:1076393. doi: 10.3389/fnagi.2022.1076393 (PMC9889977; doi:10.3389/fnagi.2022.1076393)
Supplement: Supplementary file 1 [file Table_1.docx]

Supplementary Material

Table S1: Difference between the Segments in the Young and in the Elderly Cohort with p-Values Computed with Repeated-Measures ANOVA

| **Parameter** | **Time-point 1 (T1)** | **Time-point 2 (T2)** | **Difference T2 - T1 Young Cohort** | **p-Value Young Cohort** | **Difference T2 - T1 Elderly Cohort** | **p-Value Elderly Cohort** |
| --- | --- | --- | --- | --- | --- | --- |
| *Aperiodic Parametrization* | | | | | | |
| **Exponent**  (µV^2^/Hz) | preLOC | LOC | 0,062 [-0,207 0,331] | 0,636 | **0,439 [0,224 0,655]** | **<0,001** |
|  |  | postLOC | **0,471 [0,167 0,774]** | **0,004** | **0,352 [0,109 0,595]** | **0,007** |
|  | LOC | postLOC | **0,409 [0,163 0,654]** | **0,002** | -0,087 [-0,284 0,110] | 0,370 |
| **Offset**  (µV^2^) | preLOC | LOC | **0,522 [0,091 0,952]** | **0,020** | **0,534 [0,188 0,879]** | **0,004** |
|  |  | postLOC | **0,930 [0,419 1,440]** | **0,001** | 0,182 [-0,228 0,591] | 0,637 |
|  | LOC | postLOC | 0,408 [-0,036 0,853] | 0,070 | -0,352 [-0,708 0,004] | 0,053 |
| *Periodic Parametrization of the Alpha Peak (8-12Hz)* | | | | | | |
| **Center Frequency**  (Hz) | preLOC | LOC | **0,894 [0,257 1,531]** | **0,008** | **1,035 [0,525 1,546]** | **<0,001** |
|  |  | postLOC | **0,804 [0,203 1,405]** | **0,011** | **1,038 [0,557 1,520]** | **<0,001** |
|  | LOC | postLOC | -0,090 [-0,729 0,550] | 0,773 | 0,003 [-0,516 0,509] | 0,990 |
| **Adjusted Power**  (µV^2^) | preLOC | LOC | **0,258 [0,103 0,413]** | **0,002** | -0,001 [-0,125 0,123] | 0,988 |
|  |  | postLOC | **0,473 [0,228 0,718]** | **<0,001** | **0,263 [0,067 0,460]** | **0,011** |
|  | LOC | postLOC | **0,216 [0,032 0,400]** | **0,024** | **0,264 [0,116 0,412]** | **0,001** |
| **Bandwidth**  (Hz) | preLOC | LOC | **0,768 [0,315 1,221]** | **0,002** | 0,124 [-0,239 0,487] | 0,485 |
|  |  | postLOC | **1,050 [0,421 1,680]** | **0,002** | 0,416 [-0,088 0,921] | 0,101 |
|  | LOC | postLOC | 0,282 [-0,194 0,759] | 0,232 | 0,292 [-0,090 0,674] | 0,127 |
